# Impact of the Maturation of Human Primary Bone-Forming Cells on Their Behavior in Acute or Persistent *Staphylococcus aureus* Infection Models

**DOI:** 10.3389/fcimb.2016.00064

**Published:** 2016-06-21

**Authors:** Jérôme Josse, Christine Guillaume, Camille Bour, Flora Lemaire, Céline Mongaret, Florence Draux, Frédéric Velard, Sophie C. Gangloff

**Affiliations:** ^1^EA 4691 ≪Biomatériaux et Inflammation en Site Osseux ≫, Pôle Santé, Université de Reims Champagne-ArdenneReims, France; ^2^UFR Pharmacie, Pôle Santé, Université de Reims Champagne-ArdenneReims, France; ^3^UFR Odontologie, Pôle Santé, Université de Reims Champagne-ArdenneReims, France

**Keywords:** *Staphylococcus aureus*, osteoblast, cytokine, chemokine, differentiation, maturation

## Abstract

*Staphylococcus aureus* is one of the most frequently involved pathogens in bacterial infections such as skin abscess, pneumonia, endocarditis, osteomyelitis, and implant-associated infection. As for bone homeostasis, it is partly altered during infections by *S. aureus* by the induction of various responses from osteoblasts, which are the bone-forming cells responsible for extracellular matrix synthesis and its mineralization. Nevertheless, bone-forming cells are a heterogeneous population with different stages of maturation and the impact of the latter on their responses toward bacteria remains unclear. We describe the impact of *S. aureus* on two populations of human primary bone-forming cells (HPBCs) which have distinct maturation characteristics in both acute and persistent models of interaction. Cell maturation did not influence the internalization and survival of *S. aureus* inside bone-forming cells or the cell death related to the infection. By studying the expression of chemokines, cytokines, and osteoclastogenic regulators by HPBCs, we observed different profiles of chemokine expression according to the degree of cell maturation. However, there was no statistical difference in the amounts of proteins released by both populations in the presence of *S. aureus* compared to the non-infected counterparts. Our findings show that cell maturation does not impact the behavior of HPBCs infected with *S. aureus* and suggest that the role of bone-forming cells may not be pivotal for the inflammatory response in osteomyelitis.

## Introduction

Bone remodeling is a constant natural mechanism involving the coordinated effort of two major cell populations: osteoblasts and osteoclasts. Osteoblasts form bone matrix and regulate the bone-resorbing activity of osteoclasts. Originally arising from pluripotent mesenchymal stem cells, bone-forming cells differentiate progressively in several stages and become osteoblasts that, after their terminal differentiation, are fully mature (Aubin, 2001). Each step of the osteoblastic lineage plays a specific role. For example, synthesis of extracellular matrix is mainly carried out by pre-osteoblasts and immature osteoblasts whereas mature osteoblasts are more involved in the mineralization of the extracellular matrix (Komori, 2006). Several markers of osteoblast development are expressed during their maturation. These include alkaline phosphatase, type I collagen and osteopontin, which are important for controlling bone matrix deposition and mineralization. Mature osteoblasts also produce regulators of matrix mineralization at the end of maturation such as osteocalcin and bone sialoprotein (Marie, 2008). In *in vitro* culture models, osteoblast differentiation, and maturation are mainly performed in a specific osteogenic medium (OM) containing ascorbic acid, β-glycerophosphate, and dexamethasone (Brauer et al., 2016).

There is growing evidence that osteoblasts may have additional functions with regard to cytokine production in diseases such as rheumatoid arthritis and osteoarthritis (Lisignoli et al., 2002). Osteomyelitis is a damaging bone infection that can lead to sequelae and death if left untreated and mostly results from hematogenous spreading from distant infected tissues during childhood. In adulthood, surgical procedures are increasingly responsible for direct contamination of bone tissues, especially when medical devices are used (Montanaro et al., 2011). *Staphylococcus aureus*, (*S. aureus)*, a gram-positive bacterium, is one of the most incriminated pathogens in osteomyelitis and implant-associated infections (Lowy, 1998). In the past, *S. aureus* was mostly considered to be an extracellular pyogenic pathogen but it is now known to be internalized and to persist in non-professional phagocytes while being protected from the immune system (Löffler et al., 2014). The internalization of *S. aureus* has been mainly observed in endothelial cells, lung epithelial cells, mesenchymal stem cells, and osteoblasts (Ogawa et al., 1985; Hudson et al., 1995; Jarry and Cheung, 2006; Josse et al., 2014). Moreover, osteocytes, which are the last step in osteoblast differentiation, are also able to internalize *S. aureus* (Reilly et al., 2000). *S. aureus* osteomyelitis therefore develops very often from acute infection to a state of chronicity despite the use of antimicrobial treatments. This leads to a wide spectrum of events ranging from damage to single cells to a delay in bone regeneration or excessive inflammatory response. Three major types of harmful responses for bone tissue can be triggered (reviewed in Josse et al., 2015). First, *S. aureus*-infected osteoblasts die through the activation of both apoptosis and necrosis. Second, *S. aureus* can induce the expression and the release of cytokines and chemokines. Third, among these cytokines, *S. aureus* can promote the activation and formation of osteoclasts notably via an increase in RANKL production by osteoblasts. Together, all these events can result in a massive inflammatory bone loss (Lew and Waldvogel, 2004). However, to date, the influence of the maturation stage of osteoblasts in such a setting has remained elusive.

The aim of this study was therefore to investigate whether the degree of maturation of human primary bone-forming cells (HPBCs) can impact their interaction with *S. aureus*. We cultured HPBCs in a standard growth medium to maintain their heterogeneity and in a dexamethasone-free osteogenic growth medium to obtain a population mostly composed of cells expressing maturation markers. Moreover, two models of HPBC interaction with live *S. aureus* were used in order to mimic the onset of early and persistent infection. Taken together, our data show that dexamethasone-free OM can lead to the maturation of HPBCs. Moreover, we demonstrate that the internalization and intracellular survival of *S. aureus* and *S. aureus*-related cell death are not influenced by the degree of maturation of HPBCs. Investigation of their cytokine and chemokine expression revealed some influence of their degree of maturation but no variation in relative protein release was observed.

## Materials and methods

### Bacteria culture

*S. aureus* 8325-4, a wild-type laboratory strain (prophage-free NCTC 8325), was a generous gift from T.J. Foster (Department of Microbiology, Trinity College, Dublin, Ireland). Bacteria were maintained at −80°C and streaked onto trypticase soy broth (TSB) agar plates (BioMérieux) to obtain single colonies. After overnight growth in TSB medium at 37°C, *S. aureus* was re-suspended in TSB at a starting OD (620 nm) of 0.1 and cultured at 37°C up to an exponential growth phase (OD_620_ = 0.4–0.6). The bacteria were then washed and resuspended in sterile Dulbecco's Phosphate Buffered Saline (DPBS, Gibco) for the interaction experiments described below.

### Cell culture and maturation

HPBCs used in this study were obtained from patients' femoral bone explants and prepared as described previously (Braux et al., 2011; Brun et al., 2014). Bone explants were obtained from the femoral heads of 11 patients in the orthopedic and traumatology department of the University Hospital Center in Reims, France. Samples were collected after written informed consent had been given by the donors following the guidelines approved by the University Hospital Center institutional review board. The explants were cut into small pieces, washed in DPBS four times for 5 min, digested in a solution of 0.5% trypsin, 5.3 mM EDTA (Life Technologies), and then in type II collagenase (1.4 mg.mL^−1^, Sigma Aldrich). The fragments obtained were thereafter placed in 25 cm^2^ culture flasks containing Dulbecco's Modified Eagle Medium (DMEM, Gibco) supplemented with 20% FCS (Dutscher) and 1% antibiotic solution PenStrep^®^ (Gibco), then incubated at 37°C in a 5% CO_2_ humidified atmosphere. Cells were amplified for four passages and then divided into two cell populations. The first population was cultured in the previous medium containing only 10% FCS and considered as standard medium (SM) and the second one was cultured in SM medium enriched with ascorbic acid (50 μM, Gibco) and β-glycerophosphate (10 μM, Sigma Aldrich), hereafter named osteogenic medium (OM). To avoid the anti-inflammatory effects of dexamethasone regularly used in OM (Wright and Friedland, 2004), ours was dexamethasone-free. Upon the fifth passage, cells were seeded at a density of 1 × 10^4^ cells per cm^2^ in 24-well culture plates (BD Falcon) and cultured up to 28 days in each medium to assess the osteogenic differentiation model. For fluorescent staining, cells were seeded on glass coverslips or for scanning electron microscopy on Thermanox^®^ coverslips (Thermo Scientific). LPS 0111:B4 (1 μg.mL^−1^) (Sigma Aldrich) was used to confirm the cells' ability to respond to a pro-inflammatory stimulus.

### Set of interaction between HPBCs and *S. aureus*

Infection experiments were performed on both cell populations after 14 days of culture of HPBCs. Confluent cell cultures were washed with DPBS and incubated overnight with 1 mL of respective media without antibiotics. The following day, cells were washed with DPBS, SM, or OM without antibiotics (1 mL/well) was renewed and bacteria were added. Cell count per well was determined to optimize the multiplicity of infection (MOI) in order to obtain 10:1 and 30:1 (*S. aureus*: cell). Interactions were performed according to two models defined as acute and persistent infections.

The acute infection model, representing the outbreak of an infection, was performed as previously described (Josse et al., 2014). It allows extracellular and intracellular bacteria to interact with cells. Briefly, infections were performed between bacteria and HPBCs for 1, 3, 6, and 9 h at MOI of 10:1 and 30:1 (*S. aureus*/cell).

The model of persistent infection adapted from Wright and Friedland (2002) is classically used to investigate the impact of chronic infection in osteoblasts. After 1 h of interaction between bacteria and cells (MOI of 10:1 and 30:1), co-cultures were washed twice with DPBS and then incubated with the appropriate growth medium containing 100 μg.mL^−1^ gentamycin for additional incubations to reach 9, 24, and 48 h of interaction. Gentamycin is an antibiotic that does not diffuse inside HPBCs in 48 h and so is active only on extracellular bacteria (Wilson et al., 1982). This model makes it possible to examine the impact of solely intracellular bacteria.

### Intracellular counting

At the end of each interaction time in the model of acute infection, co-cultures were washed three times with DPBS and incubated 1 h with growth medium containing 100 μg.mL^−1^ gentamycin at 37°C in a 5% CO_2_ humidified atmosphere. Then, cells were washed three times with DPBS and 0.1% Triton X-100 solution was added to each well to harvest intracellular bacteria. Appropriated dilutions of the lysates were plated in triplicate on TSA plates for determination of the recovered colony forming units (CFU) after incubation for 18 h at 37°C.

For the model of persistent infection, co-cultures were processed in the same way and washed three times with DPBS before the numbers of intracellular bacteria were determined as described above.

### Alkaline phosphatase activity

Alkaline phosphatase activity was determined by using the precipitating substrate BCIP^®^/NBT (SigmaFast™, Sigma). At various time intervals (7, 14, 21, and 28 days), cell cultures were rinsed twice with DPBS and fixed with 4% paraformaldehyde in DPBS. Then, wells were rinsed twice with DPBS and incubated for 10 min with BCIP^®^/NBT. Thereafter, wells were rinsed twice with DPBS and dried in order to reveal by imaging the precipitation in the entire wells (Scanner Epson Perfection 1660 Photo).

### Alizarin red staining

At 7, 14, 21, and 28 days, cells were rinsed twice with DPBS and fixed with 4% paraformaldehyde in DPBS. Then, wells were rinsed twice with DPBS and distilled water. Cell cultures were incubated for 10 min with alizarin red staining solution (Sigma). At the end of the incubation period, wells were rinsed twice with DPBS and dried in order to image the entire wells (Scanner Epson Perfection 1660 Photo).

### Type I collagen and osteocalcin immunofluorescent staining

Twenty-one-day cell cultures were rinsed twice with DPBS and then fixed with 4% paraformaldehyde (Agar Scientific LTD) in DPBS containing 0.1% Triton X-100 (Prolabo) for 5 min at room temperature. After DPBS rinsing and 3% bovine serum albumin (BSA, Sigma Aldrich) saturation for 1 h, samples were incubated for 1 h with anti-human type I collagen antibodies (clone H-197, Santa-Cruz) or with anti-human osteocalcin antibodies (clone 190125, R&D Systems) (diluted at 1/400 and 1/50 respectively). After another BSA saturation step, samples were incubated with biotinylated horse anti-rabbit or goat anti-mouse secondary antibodies (diluted at 1/50, Vector) for 30 min, rinsed in DPBS and incubated in a solution of streptavidine-Alexafluor^®^488 (diluted at 1/200, Molecular Probes) for 30 min and rinsed in DPBS. After rinsing with dH_2_O, cell nuclei were counterstained for 5 min with DAPI (Invitrogen). These cells were mounted on glass coverslips (Fluorescent Mounting Medium Dako) and then visualized with a Zeiss Axiovert 200 M inverted microscope (Carl Zeiss).

### LDH measurement

Lactate dehydrogenase (LDH) activity in cell supernatant is relevant for evaluating cell membrane damage. At the end of each time period for both models of infection, LDH activity was assessed on conditioned cell culture supernatants (Cytotoxicity detection kit plus^®^, Roche Diagnostics) following the manufacturer's instructions. Absorbance was read at 592 nm with correction of non-specific background at 700 nm.

### Enzyme-linked immunosorbent assay

Pro-inflammatory cytokines (IL-1β and IL-6), chemokines (CCL2, CXCL1, and CXCL8), and OPG concentrations in conditioned supernatants were measured by ELISA, according to the recommended protocol (Duoset^®^, R&D Systems). Controls were composed of the non-stimulated cells and media alone.

### Real-time PCR analysis

At the end of each infection period for both infection models, total RNA was extracted from HPBCs by using the MasterPure™ RNA Purification Kit (Epicentre^®^ Biotechnologies) in accordance with the manufacturer's instructions. RNA quantity and purity of the samples were assessed using a Nanodrop^®^ (Thermo Scientific). All sample OD_260_/OD_280_ ratios were between 1.95 and 2.05. Thereafter, RNA was reverse-transcribed into cDNA using a High-Capacity cDNA Reverse Transcription kit following the manufacturer's instructions (Applied Biosystems). The cDNA product was amplified by real-time PCR (Step One Plus^®^, Applied Biosystems) and the mRNA levels for osteogenic genes, inflammatory genes, and internal control *HPRT-1* were determined using the double strand-specific Power SYBR^®^ Green dye system (Applied Biosystems). Primer sequences of all genes were determined with the Universal Probe Library Assay Design Center (Roche Applied Science). Primer efficiency was also measured (Table 1). Data analysis was performed with the Step One software v2.3 (Applied Biosystems). Results were calculated in accordance with the 2^−ΔΔCt^ method and are presented as fold ratios.

**Table 1:** **Primer nucleotide sequence for each primer couple**.

**Target gene**	**Sequences**	
	**Forward primer**	**Reverse primer**
*COL1A1*	5′-GGGATTCCCTGGACCTAAAG-3′	5′-GGAACACCTCGCTCTCCAG-3′
*SPARC*	5′-TTCCCTGTACACTGGCAGTTC-3′	5′-AATGCTCCATGGGGATGA-3′
*SPP1*	5′-GAGGGCTTGGTTGTCAGC-3′	5′-CAATTCTCATGGTAGTGAGTTTTCC-3′
*IBSP*	5′-GAGGGCTTGGTTGTCAGC-3′	5′-CAATTCTCATGGTAGTGAGTTTTCC-3′
*BGLAP*	5′-TGAGAGCCCTCACACTCCTC-3′	5′-ACCTTTGCTGGACTCTGCAC-3′
*IL-1β*	5′-CTGTCCTGCGTGTTGAAAGA-3′	5′-TTGGGTAATTTTTGGGATCTACA-3′
*IL-6*	5′-GGAACAAGCCAGAGCTGTG-3′	5′-GGCTGGCATTTGTGGTTGG-3′
*TNF-α*	5′-CAGCCTCCTCTCCTTCTTGAT-3′	5′-GCCAGAGGGCTGATTAGAGA-3′
*CCL2*	5′-AGTCTCTGCCGCCCTTCT-3′	5′-GTGACTGGGGCATTGATTG-3′
*CXCL1*	5′-TCCTGCATCCCCCATAGTTA-3′	5′-CTTCAGGAACAGCCACCAGT-3′
*CXCL8*	5′-AGACAGCAGAGCACACAAGC-3′	5′-CTCCTTGGCAAAACTGCAC-3′
*RANKL*	5′-TGATTCATGTAGGAGAATTAAACAGG-3′	5′-GATGTGCTGTGATCCAACGA-3′
*OPG*	5′-GAAGGGCGCTACCTTGAGAT-3′	5′-GCAAACTGTATTTCGCTCTGG-3′
*HPRT-1*	5′-TGACCTTGATTTATTTTGCATACC-3′	5′-CGAGCAAGACGTTCAGTCCT-3′

### Scanning electron microscopy (SEM)

SEM was performed for acute infection. After 9 h of co-culture, samples were rinsed twice with DPBS and then fixed with 2.5% glutaraldehyde in DPBS for 1 h at room temperature. After graded ethanol dehydration the samples were immersed in HMDS for 5 min, air-dried at room temperature and sputtered with a thin gold–palladium film under a JEOL ion sputter JFC 1100 (JEOL). Cells were observed with a LaB6 electron microscope (JEOL JSM-5400 LV).

### Graphical representation of data and statistics

Each experiment was performed on cells from 4 of 11 independent donors. Each pool of cells consisted of cells grown in both SM and OM. Data are presented as whisker plots: black spots represent maximum and minimum values, black bars represent 1st and 9th decile, the bottom and top of the box are the 1st and 3rd quartiles, and the red band inside the box stands for the median. Gray box plots were used for HPBCs cultured in SM (SM-HPBCs) and black box plots were used for HPBCs cultured in OM (OM-HPBCs). Owing to a lack of normal distribution of the assessed variables due to the small number of donors, non-parametric exact Wilcoxon-Mann-Whitney tests with the *p*-value fixed at 0.05 were carried out to determine the significance of the results (StatXact 7.0, Cytel Inc.).

## Results

### Cell maturation is effective even without dexamethasone

**Figure 1:**
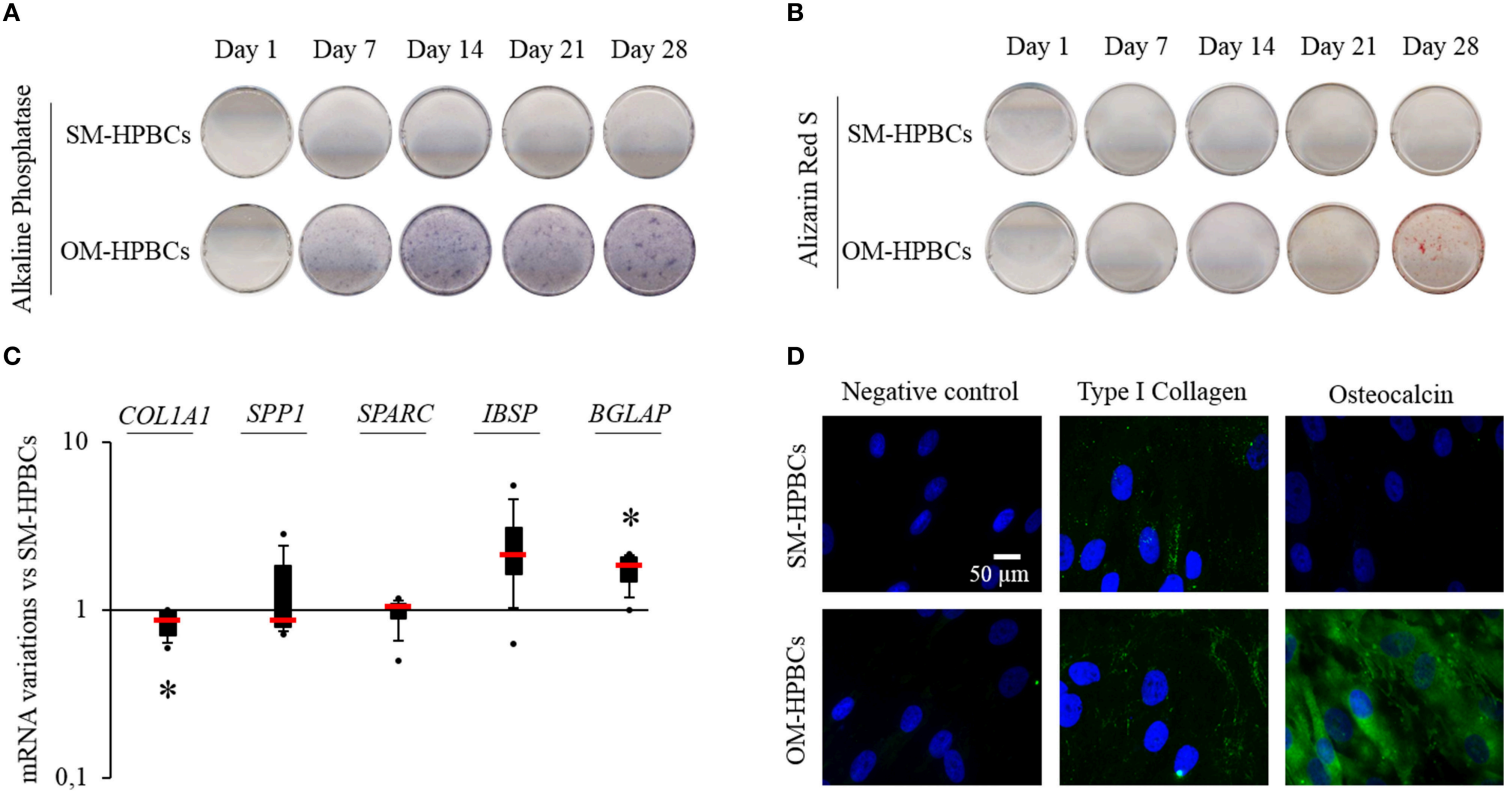
**Osteogenic differentiation (A–D). (A)** Representative photographs of alkaline phosphatase staining of SM- and OM-HPBCs after 7, 14, 21, and 28 days of culture, **(B)** alizarin red staining of SM- and OM-HPBCs after 7, 14, 21, and 28 days of culture, and **(C)** expression of osteogenic genes by OM- compared to SM-HPBCs after 14 days of culture (mRNA were evaluated by RT-qPCR analysis, data were shown as specific variation in mRNA using the 2^−ΔΔCt^ method (*HPRT-1* was used as internal control), ^*^ means *p* < 0.05 vs. SM-HPBCs). **(D)** Representative micrographs of 21-day immunofluorescent staining of cultured cells: type I collagen and osteocalcin were visualized in green with Alexafluor^®^488. Nuclei were counterstained in blue by DAPI. Scale bar = 50 μm. Experiments were performed on four independent donors.

To investigate whether maturation may impact the behavior of HPBCs during *S. aureus* interaction, two populations of HPBCs at different stages of maturation were established. Maturation of osteoblasts is commonly performed by using OM containing dexamethasone. However, the anti-inflammatory molecule dexamethasone impacts cell signaling and would interfere in the readout of the studied mediators in our experiments. Therefore, specific osteoblast markers were assessed on both HPBCs population during their culture to assess the effectiveness of dexamethasone-free OM to promote osteoblastic maturation as compared to SM (Figure 1).

Cells cultured in SM, hereafter named SM-HPBCs, presented a very low alkaline phosphatase activity and the alizarin red staining remained negative up to day 28 of culture (Figures 1A,B). Cells cultured in dexamethasone-free OM, hereafter named OM-HPBCs, exhibited a high alkaline phosphatase activity observed from day 14 to 28 as well as a positive alizarin red staining after day 28 of culture (Figures 1A,B). The mRNA expressions of five major bone extracellular matrix proteins were also analyzed to evaluate the commitment in osteogenic differentiation for both populations of V. As seen in Figure 1C after 14 days of culture, *COL1A1* expression was statistically lower for the OM-HPBCs compared to the expression by SM-HPBCs. No difference in *SPARC* and *SPP1* expressions could be seen. *IBSP* expression appeared upregulated and *BGLAP* was significantly increased in OM-HPBCs as compared to SM-HPBCs.

Immunofluorescence staining at day 21 confirmed the difference between the cultured cells (Figure 1D). Low production of type I collagen and absence of osteocalcin were observed for SM-HPBCs. On the contrary, type I collagen extracellular fibers as well as the presence of osteocalcin could be detected for OM-HPBCs.

These results suggested that at a given time, the culture of HPBCs in a dexamethasone-free OM promoted a higher production of specific mature osteoblast markers compared to HPBCs cultured in SM. Therefore, OM-HPBCs may be considered as more mature than SM-HPBCs.

### *S. aureus* can survive inside HPBCs

**Figure 2:**
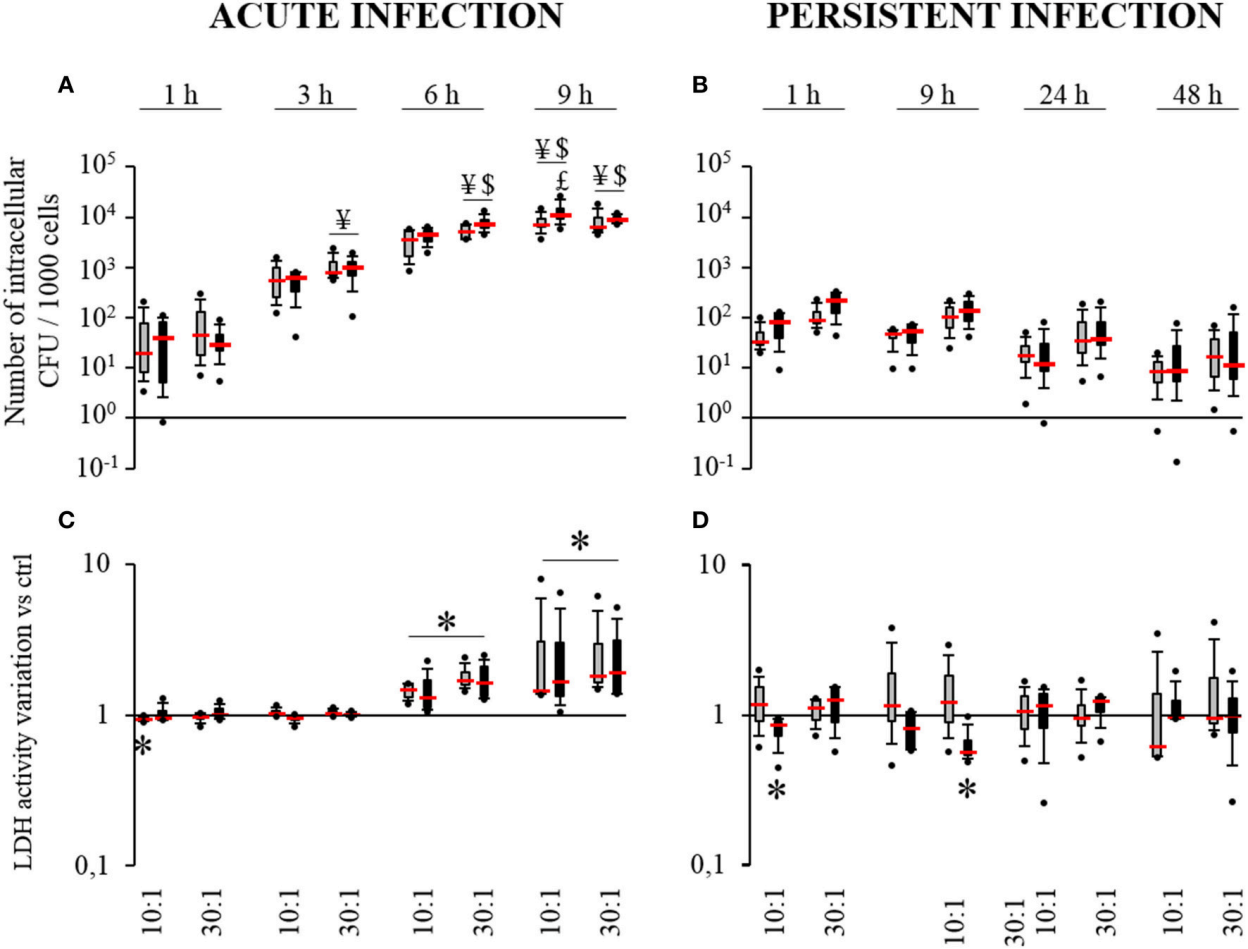
**Internalization (A) and survival of *S. aureus* (B) and effect of *S. aureus* on cell death (C,D)**. SM- (gray boxes) and OM-HPBCs (black boxes) were exposed to viable *S. aureus* (MOI of 10:1 and 30:1) for 1, 3, 6, and 9 h in acute infection **(A,C)** and for 1, 9, 24, and 48 h in persistent infection **(B,D)**. For internalization **(A)** and survival of *S. aureus* **(B)**, the number of intracellular bacteria was determined after serial plating. ¥ means *p* < 0.05 compared to value at 1 h for the same MOI, $ means *p* < 0.05 compared to value at 3 h for the same MOI, $ means *p* < 0.05 compared to value at 6 h for the same MOI. For cell viability **(C,D)**, LDH activity was measured in cell supernatants. Data are shown as specific variation in optical density compared to optical density of relative non-infected cells. ^*^ means *p* < 0.05 compared to non-infected cells. Experiments were performed on four independent donors.

Acute and persistent infection models were used in order to investigate the impact of HPBC maturation on the internalization and the survival of *S. aureus* inside HPBCs. As seen in the acute infection model (Figure 2A), the average number of intracellular bacteria was under 120 bacteria per 1000 cells after 1 h of interaction, irrespective of the cell differentiation. This figure regularly increased with time up to 9 h. After 9 h of interaction, the average number of internalized live bacteria reached at least 8350 bacteria per 1000 cells.

In the persistent infection model (Figure 2B), the number of intracellular *S. aureus* seemed to decrease slightly from 1 to 48 h irrespective of the cell differentiation. Intracellular staphylococci survived inside the cells until 48 h with an average count between 10 and 45 bacteria per 1000 cells depending on the MOI. For each time, the number of bacteria was systematically higher with the MOI of 30:1 than with the MOI of 10:1.

Comparing both models at 9 h post infection, at least a 2 log reduction was observed in the number of intracellular bacteria in the cells after persistent infection as compared to acute infection (Figures 2A,B).

**Figure 3:**
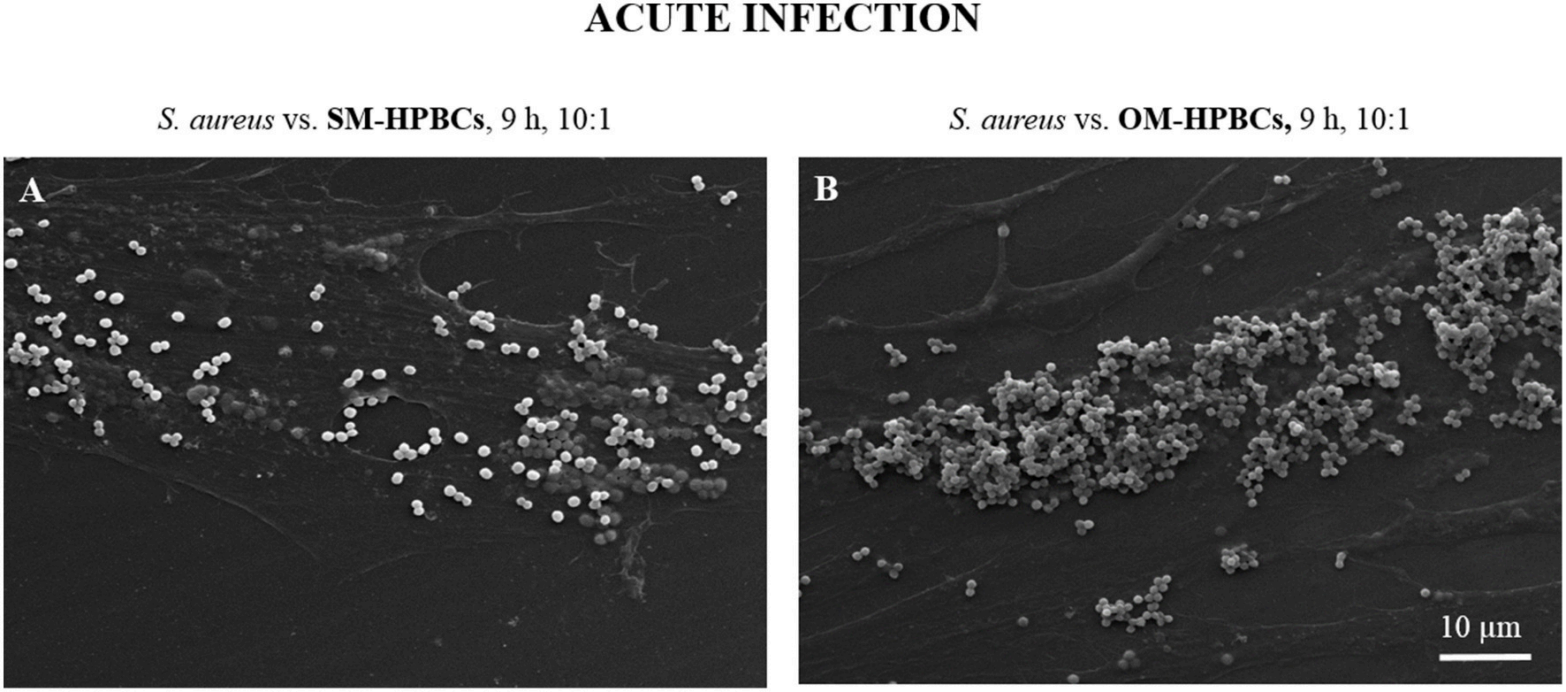
**Representative SEM micrographs of interaction between *S. aureus* and SM- (A) or OM-HPBCs (B) after 9 h at the MOI of 10:1 in acute infection model**. Magnification x1000.

Similar results for internalization and survival of *S. aureus* were observed in both SM- and OM-HPBCs, suggesting that HPBC maturation did not influence these phenomena, though a difference in bacterial adhesion was observed between SM- and OM-HPBCs (Figure 3).

Nevertheless, the ability of HPBCs to contain viable intracellular bacteria was strongly influenced by the presence of extracellular bacteria.

### *S. aureus* induces cell death

To determine whether cell maturation influences *S. aureus*-induced cell death, we measured the release of LDH from both SM and OM-HPBCs. In the acute infection model, a low but significant decrease in LDH activity was observed at 1 h for SM-HPBCs. After 6 h of interaction, significant increases in LDH activity were detected in the presence of *S. aureus* at the MOIs of 10:1 or 30:1 for both SM and OM-HPBCs (Figure 2C). At 9 h post-infection a greater and significant LDH release was observed without any MOI effects (10:1 or 30:1).

As for the persistent infection model, the release of LDH was more variable between each time point, with the level of LDH sometimes even lower for the infected cells, like for OM-HPBCs at 1 and 9 h post-infection (Figure 2D). Apart from that, the quantity of LDH released by both populations of HPBCs was statistically similar in the presence of *S. aureus* from 1 to 48 h.

Similar results were observed for both SM- and OM-HPBCs, suggesting that HPBC maturation has no influence in the protection against cell death caused by *S. aureus*. However, the accumulation of extracellular *S. aureus* increased cell death in the acute infection model.

### *S. aureus*-related production of pro-inflammatory cytokines by HPBCs

The pro-inflammatory cytokines IL-1β, IL-6, and TNF-α are potent activators of osteoclastogenesis. Their presence in the bone microenvironment promotes inflammatory processes and the recruitment of pre-osteoclasts and their activation. To further characterize the impact of *S. aureus* on SM- and OM-HPBCs, the expression of these cytokines was analyzed.

**Figure 4:**
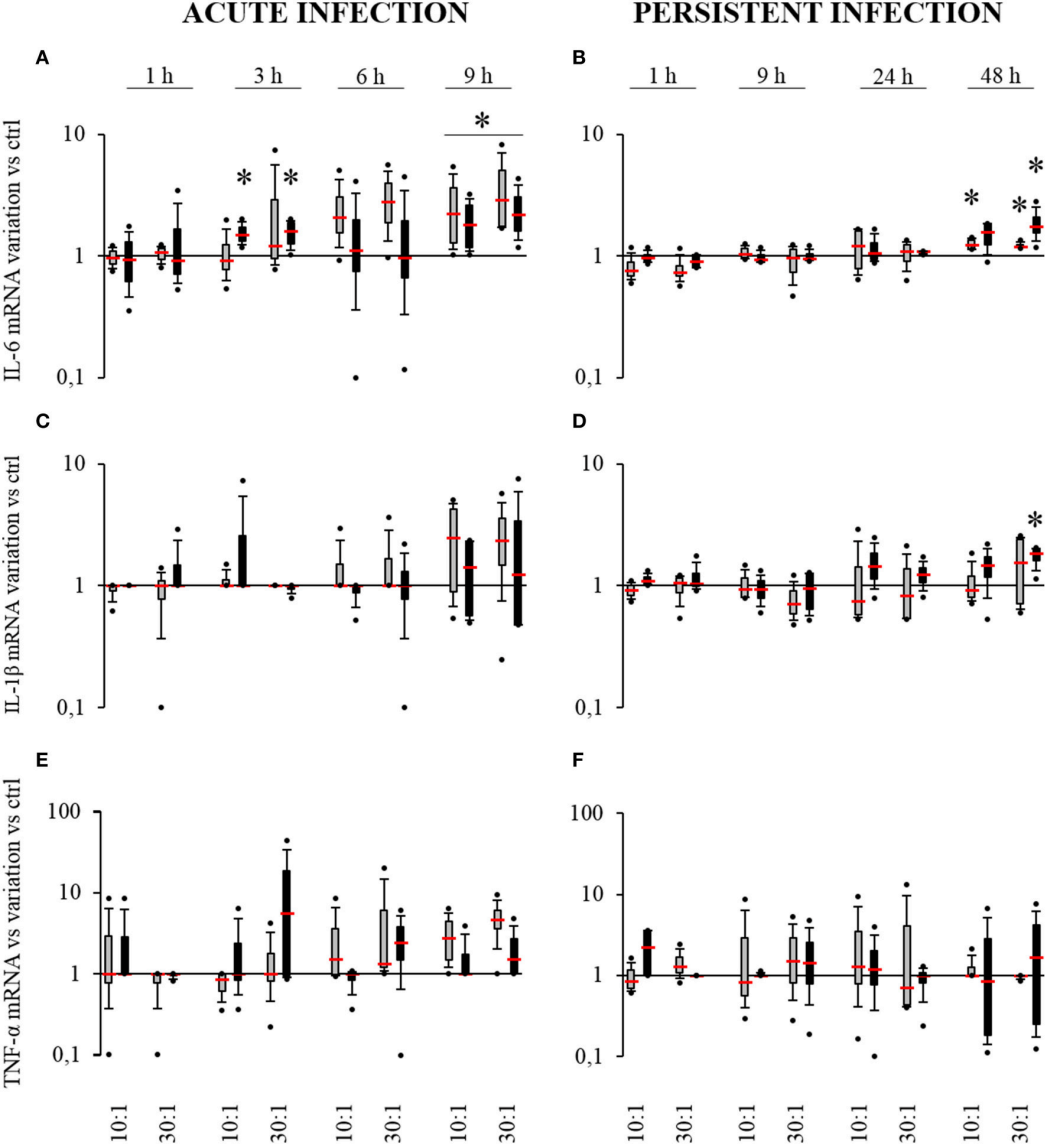
**Effect of *S. aureus* on *IL-6* (A,B), *IL-1β* (C,D), and *TNF-α* (E,F) mRNA expressions by SM- (gray boxes) and OM-HPBCs (black boxes)**. Cells were exposed to viable *S. aureus* (MOI of 10:1 and 30:1) for 1, 3, 6, and 9 h in acute infection **(A,C,E)** and for 1, 9, 24, and 48 h in persistent infection **(B,D,F)**. Expression of *IL-6, IL-1β*, and *TNF-α* mRNA was evaluated by RT-qPCR analysis. Data are shown as specific variation of mRNA compared to relative non-infected cells using the 2^−ΔΔCt^ method (*HPRT-1* was used as internal control). ^*^ means *p* < 0.05 vs. relative non-infected cells for each time period. Experiments were performed on four independent donors.

In the acute infection model, *S. aureus* induced an increase in *IL-6* expression by SM-HPBCs that started at 6 h. At 9 h post-infection, this overexpression in infected cells corresponded to a 2.7-fold increase for the MOI of 10:1 and a 3.9-fold increase for the MOI of 30:1. For OM-HPBCs, *S. aureus* also induced significant increases in *IL-6* expression at 3 h with an average of 1.5-fold for both MOIs. At 9 h post-infection a two-fold overexpression for the MOI of 10:1 and of 2.5-fold for the MOI of 30:1 were observed (Figure 4A). No significant variations were observed for *IL-1β* and *TNF-α* expression in the acute infection model even if a trend for increase was observed for both the cytokines mRNAs in the presence of *S. aureus* after 9 h (Figures 4C,E).

Of note, *IL-1β*, *IL-6*, and *TNF-α* expressions were up-regulated from 3 to 9 h by both SM and OM-HPBCs stimulated with LPS (Supplementary Figures 1A,C,E).

In the persistent infection model, a low but significant 1.2-fold increase in *IL-6* mRNA expression was observed for infected SM-HPBCs after 48 h of interaction at both 10:1 and 30:1 MOIs. The same profile was seen with OM-HPBCs, with a significant 1.9-fold increase for the MOI of 30:1 (Figure 4B). Concerning *IL-1β*, *S. aureus* induced a significant 1.7-fold increase in mRNA expression in OM-HPBCs after 48 h of infection (Figure 4D). No significant variations in *TNF-α* expression were observed in the model of persistent infection (Figure 4F).

Of note, *IL-1β* and *IL-6* expressions were up-regulated in both SM- and OM-HPBCs stimulated with LPS until 48 h whereas *TNF-α* expression only increased at 1 and 9 h (Supplementary Figures 1B,D,F).

To further investigate the protein release that could correlate with the significant mRNA expression modulations, IL-6 and IL-1β protein release was measured. Concerning IL-6 (Table 2), the protein release was detected in supernatants of the cells of all donors after 3 h in the acute model of infection and after 9 h in the persistent infection one. Thereafter, the release of IL-6 by both cell types increased regularly in both acute and persistent infection models and remained within the levels of the non-infected control HPBCs. However, LPS stimulation induced an upregulation of IL-6 when compared to the control cells (Supplementary Table 1). No protein release of IL-1β by HPBCs was detected regardless of the model of infection, even after stimulation with LPS (data not shown).

**Table 2:** **Effect of *S. aureus* on IL-6 protein release by SM-HPBCs (white columns) and OM-HPBCs (gray columns)**.

**Acute infection**																								
**IL-6**	**1 h**						**3 h**						**6 h**						**9 h**					
**pg.mL**−1	**Ctrl**		**10:1**		**30:1**		**Ctrl**		**10:1**		**30:1**		**Ctrl**		**10:1**		**30:1**		**Ctrl**		**10:1**		**30:1**	
Max	97	209	97	136	75	119	429	442	397	317	375	337	702	604	560	498	647	630	927	924	892	893	894	843
Median	22	5	14	12	15	7	99	73	72	61	111	57	254	153	328	231	347	242	671	717	671	718	667	702
Min	–	–	–	–	–	–	14	29	31	18	30	19	113	120	105	94	173	78	471	571	442	527	419	459
**Persistent infection**																								
**IL-6**	**1 h**						**9 h**						**24 h**						**48 h**					
**pg.mL**−1	**Ctrl**		**10:1**		**30:1**		**Ctrl**		**10:1**		**30:1**		**Ctrl**		**10:1**		**30:1**		**Ctrl**		**10:1**		**30:1**	
Max	2097	1947	1688	2136	1983	1939	3324	3242	2670	3212	3250	3152	4523	4852	3943	5095	4778	5023	5011	6250	4284	5773	4778	6155
Median	726	895	714	781	683	750	2445	2540	1682	2409	1918	2213	3712	3422	3068	3047	3315	3047	3939	4066	3170	3739	3358	3630
Min	24	–	105	17	–	–	583	82	521	73	424	54	779	85	738	121	612	82	833	94	750	119	810	114

*Cells were exposed to viable S. aureus (MOI of 10:1 and 30:1) for 1, 3, 6, and 9 h in acute infection and for 1, 9, 24, and 48 h in persistent infection. Data are presented as minimal value, median value, and maximal value from four independent donors (– means undetermined)*.

In summary, *S. aureus* induced low but statistically significant increases in mRNA expression of IL-6 in both SM- and OM-HPBCs and a small increase in IL-1β mRNA at 48 h post-infection in OM-HPBCs. These results suggest that HPBC maturation did not influence cytokine expression. In our infection models, we also found that the relative protein release by SM- and OM-HPBCs was not influenced by the presence of *S. aureus* even if cells of all our donors were responsive to LPS stimulation.

### *S. aureus*-related production of chemokines by HPBCs

Chemokines are well known for playing a role in antibacterial defense notably through the recruitment of neutrophils and monocytes/macrophages. To further investigate how HPBC maturation modulates the inflammatory response of infected cells, the expression and the release of CCL2, CXCL1, and CXCL8 by SM- and OM-HPBCs infected with *S. aureus* was evaluated.

**Figure 5:**
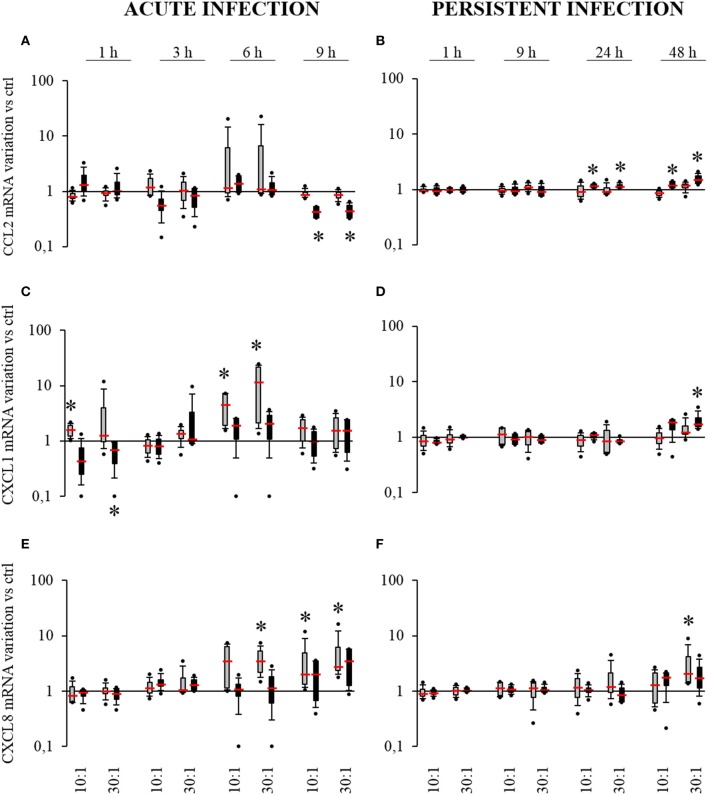
**Effect of *S. aureus* on *CCL2* (A,B), *CXCL1* (C,D), and *CXCL8* (E,F) mRNAs expressions by SM- (gray boxes) and OM-HPBCs (black boxes)**. Cells were exposed to viable *S. aureus* (MOI of 10:1 and 30:1) for 1, 3, 6, and 9 h in acute infection **(A,C,E)** and for 1, 9, 24, and 48 h in persistent infection **(B,D,F)**. Expressions of *CCL2, CXCL1*, and *CXCL8* mRNA were evaluated by RT-qPCR analysis. Data are shown as specific variation in mRNA compared to relative non-infected cells using the 2^−ΔΔCt^ method (*HPRT-1* was used as internal control). ^*^ means *p* < 0.05 vs. relative non-infected cells for each time period. Experiments were performed on four independent donors.

In acute infection, no variation in *CCL2* mRNA expression was measured for infected SM-HPBCs compared to their non-infected counterparts. For infected OM-HPBCs, a significant two-fold decrease in *CCL2* expression was observed at MOIs of 10:1 and 30:1 after 9 h (Figure 5A). Focusing on *CXCL1* expression (Figure 5C), at 6 h post-infection a 4.5-fold increase in *CXCL1* expression was observed for SM-HPBCs at the MOI 10:1 as well as a 12.3-fold increase for MOI 30:1. At 9 h post infection, the expression was decreased but still in the upper range as compared to the basal expression by uninfected SM-HPBCs. Levels of *CXCL1* mRNA appeared higher for infected cells at 6 h than in the uninfected cells though there were no significant differences for OM-HPBCs (Figure 5C). For *CXCL8*, SM-HPBCs interacting with *S. aureus* exhibited significant increases in mRNA expression (Figure 5E). At 6 h, a significant four-fold mean increase was observed for the MOI of 30:1. At 9 h, *S. aureus* significantly upregulated the mean expression of *CXCL8* by 4.2-fold and 5.9-fold for the MOIs of 10:1 and 30:1, respectively. For OM-HPBCs, *S. aureus* did not significantly impact *CXCL8* expression. Nevertheless, a trend for an increase was observed after 9 h interaction between OM-HPBCs and *S. aureus* (Figure 5E).

Of note, *CCL2, CXCL1*, and *CXCL8* expression was up-regulated by both SM and OM-HPBCs from 3 to 9 h after stimulation with LPS (Supplementary Figures 2A,C,E).

In the persistent infection model, no significant variations in *CCL2* by SM-HPBCs were observed (Figure 5B). Conversely OM-HPBCs showed low but significant increases in *CCL2* expression at both 24 h and 48 h. A 1.2-fold mean increase was observed for both MOIs of 10:1 and 30:1 at 24 h. At 48 h, *S. aureus*-induced mean increases of 1.2-fold and 1.6-fold for the MOIs of 10:1 and 30:1 respectively (Figure 5B). Concerning *CXCL1* expression, no significant variations were observed for SM-HPBCs (Figure 5D). For OM-HPBCs, a significant mean increase in *CXCL1* expression by 2.1-fold was observed at 48 h for the MOI of 30:1 (Figure 4D). For *CXCL8* expression, slight increases were detected after 48 h in the presence of *S. aureus* with a significant 3.6-fold mean increase in SM-HPBCs infected at the MOI of 30:1 (Figure 5F). *CCL2, CXCL1*, and *CXCL8* expressions were up-regulated by both SM and OM-HPBCs stimulated with LPS until 48 h (Supplementary Figures 2B,D,F).

To further investigate the impact of *S. aureus* on HPBCs, the protein release of the three previously studied chemokines was measured.

In the acute infection model (Table 3), CCL2 and CXCL8 protein releases were detected in supernatants of the cells of all donors only after 9 h and were within the levels of the non-infected control HPBCs.

**Table 3:** **Effect of *S. aureus* on CCL2, CXCL1, and CXCL8 protein release by SM-HPBCs (white columns) and OM-HPBCs (gray columns)**.

**Acute infection**																								
**CCL2**	**1 h**						**3 h**						**6 h**						**9 h**					
**pg.mL**−1	**Ctrl**		**10:1**		**30:1**		**Ctrl**		**10:1**		**30:1**		**Ctrl**		**10:1**		**30:1**		**Ctrl**		**10:1**		**30:1**	
Max	573	1259	355	1105	405	1005	1232	2341	950	2291	665	2668	5123	8059	5609	8027	6900	7695	5877	7538	5375	5641	5142	8082
Median	–	306	–	189	–	–	–	589	–	623	–	639	407	704	589	791	809	942	3870	3310	2272	4754	1806	4443
Min	–	–	–	–	–	–	–	–	–	–	–	–	–	–	–	–	–	–	426	2444	593	1060	523	1000
**Persistent infection**																								
**CCL2**	**1 h**						**9 h**						**24 h**						**48 h**					
**pg.mL**−1	**Ctrl**		**10:1**		**30:1**		**Ctrl**		**10:1**		**30:1**		**Ctrl**		**10:1**		**30:1**		**Ctrl**		**10:1**		**30:1**	
Max	2294	1792	2188	2133	1775	1871	6525	6037	3325	5926	3738	6532	9500	10,329	8381	9352	9331	9912	13,756	11,458	11,494	11,356	12,889	11,389
Median	674	1473	556	1474	640	1465	2722	4955	2640	4999	2855	4935	6007	7154	5314	7368	5115	7487	9324	7040	7296	8226	10,915	8274
Min	–	–	–	–	–	–	1983	4102	1539	4242	2083	3942	2761	6019	2517	5989	2722	6027	2928	2008	3261	1742	4549	7633
**Acute infection**																								
**CXCL1**	**1 h**						**3 h**						**6 h**						**9 h**					
**pg.mL**−1	**Ctrl**		**10:1**		**30:1**		**Ctrl**		**10:1**		**30:1**		**Ctrl**		**10:1**		**30:1**		**Ctrl**		**10:1**		**30:1**	
Max	–	218	220	–	224	–	222	–	238	–	238	–	216	214	260	211	218	211	–	317	275	361	269	807
Median	–	–	–	–	–	–	–	–	–	–	–	–	–	198	–	97	–	99	–	259	93	286	90	273
Min	–	–	–	–	–	–	–	–	–	–	–	–	–	–	–	–	–	–	–	–	–	–	–	–
**Persistent infection**																								
**CXCL1**	**1 h**						**9 h**						**24 h**						**48 h**					
**pg.mL**−1	**Ctrl**		**10:1**		**30:1**		**Ctrl**		**10:1**		**30:1**		**Ctrl**		**10:1**		**30:1**		**Ctrl**		**10:1**		**30:1**	
Max	–	–	–	–	–	–	271	291	–	346	–	373	228	413	230	406	285	377	262	517	278	670	287	555
Median	–	–	–	–	–	–	93	92	–	97	–	98	–	110	–	106	119	102	–	149	–	121	–	132
Min	–	–	–	–	–	–	–	–	–	–	–	–	–	–	–	–	–	–	–	–	–	–	–	–
**Acute infection**																								
**CXCL8**	**1 h**						**3 h**						**6 h**						**9 h**					
**pg.mL**−1	**Ctrl**		**10:1**		**30:1**		**Ctrl**		**10:1**		**30:1**		**Ctrl**		**10:1**		**30:1**		**Ctrl**		**10:1**		**30:1**	
Max	–	–	–	–	–	–	46	–	41	–	44	–	45	220	258	530	353	528	415	707	413	690	410	687
Median	–	–	–	–	–	–	–	–	–	–	–	–	–	38	–	33	–	29	212	370	215	372	186	357
Min	–	–	–	–	–	–	–	–	–	–	–	–	–	–	–	–	–	–	–	27	35	37	32	31
**Persistent infection**																								
**CXCL8**	**1 h**						**9 h**						**24 h**						**48 h**					
**pg.mL**−1	**Ctrl**		**10:1**		**30:1**		**Ctrl**		**10:1**		**30:1**		**Ctrl**		**10:1**		**30:1**		**Ctrl**		**10:1**		**30:1**	
Max	198	262	191	240	192	236	651	461	498	470	573	457	678	521	637	481	782	496	718	570	633	551	674	613
Median	79	94	66	83	84	93	443	387	408	354	386	350	524	436	541	404	529	417	566	472	597	466	627	538
Min	–	–	–	–	–	–	–	51	–	52	–	64	27	213	–	195	–	197	50	270	35	300	53	385

*Cells were exposed to viable S. aureus (MOI of 10:1 and 30:1) for 1, 3, 6, and 9 h in acute infection and for 1, 9, 24, and 48 h in persistent infection. Data are presented as minimal value, median value, and maximal value from four independent donors (– means undetermined)*.

In the persistent model of infection (Table 3), the release of CCL2 by the HPBCs of all donors increased with time from 9 to 48 h and was within the levels of the non-infected HPBCs. Concerning CXCL8, the protein release was detected in supernatants of OM-HPBCs of all donors from 9 to 48 h and stayed within the levels of the non-infected control OM-HPBCs whereas it only occurred at 48 h for SM-HPBCs.

No *CXCL1* protein release was detected in the supernatants of the cells of any of the donors at the same time in both the acute and persistent infection models (Table 3). LPS stimulation nevertheless induced up-regulation of all the three chemokines when compared to the control cells in both models of infection (Supplementary Table 2).

Taken together, these results revealed that the infected SM-HPBCs are more able to upregulate their chemokine expression compared to the infected OM-HPBCs in the conditions of acute infection, suggesting an impact of HPBC maturation. However, we also found that the relative protein release by both SM- and OM-HPBCs was not influenced by the presence of *S. aureus* even if the cells of all our donors were responsive to LPS stimulation.

### *S. aureus*-related production of OPG and RANKL by HPBCs

**Figure 6:**
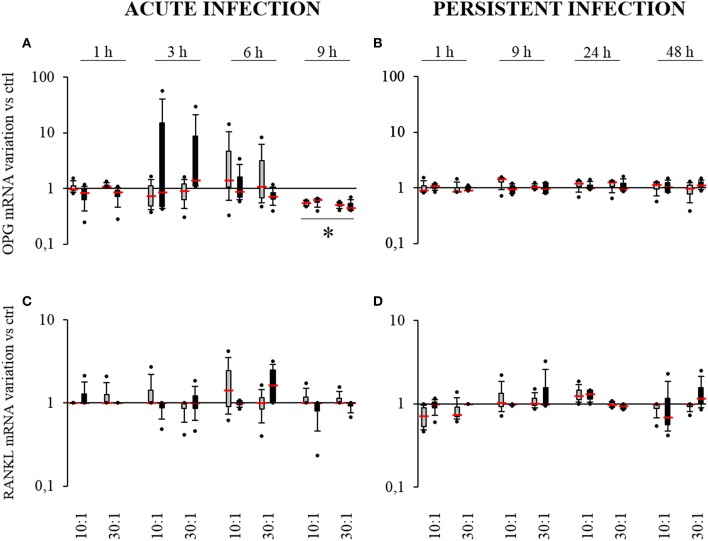
**Effect of *S. aureus* on *OPG* (A,B) and *RANKL* (C,D) mRNAs expressions by SM- (gray boxes) and OM-HPBCs (black boxes)**. Cells were exposed to viable *S. aureus* (MOI of 10:1 and 30:1) for 1, 3, 6, and 9 h in acute infection **(A,C)** and for 1, 9, 24, and 48 h in persistent infection **(B,D)**. Expressions of *OPG* and *RANKL* mRNA were evaluated by RT-qPCR analysis. Data are shown as specific variation of mRNA compared to relative non-infected cells using the 2^−ΔΔCt^ method (*HPRT-1* was used as internal control). ^*^ means *p* < 0.05 vs. relative non-infected cells for each time period. Experiments were performed on four independent donors.

Bone-forming cells can modulate osteoclastogenesis through the production of RANKL and OPG. RANKL binds to RANK on osteoclast precursors to promote their activation whereas OPG is a decoy receptor for RANKL that can decrease osteoclastogenesis. The impact of osteogenic differentiation and *S. aureus* interaction on *OPG* and *RANKL* expression was therefore evaluated (Figure 6).

In the acute infection model, for both SM- and OM-HPBCs, 9 h were required to observe a two-fold mean significant decrease in *OPG* expression for both 10:1 and 30:1 MOIs (Figure 6A). Conversely LPS stimulation induced a significant increase in *OPG* expression at 6 and 9 h with both HPBCs (Supplementary Figure 3A).

In the persistent infection model, the level of *OPG* mRNA remained equal in the presence or absence of bacteria for both HPBCs up to 48 h whereas an increase in expression was observed from 9 to 48 h when both the HPBCs were stimulated with LPS (Figure 6B, Supplementary Figure 3B). Concerning RANKL, no significant modulation of expression was observed in any condition even with LPS stimulation (Figures 6C,D, Supplementary Figures 3C,D).

To further investigate the impact of *S. aureus* on HPBCs, the protein release of OPG was measured in cell supernatants. In the acute infection model (Table 4), OPG protein release was detected in supernatants of the cells of all donors from 6 to 9 h and was within the levels of the non-infected control HPBCs. On the whole, the release by all the cells increased from 1 to 6 h and decreased at 9 h. In the persistent model of infection (Table 4), OPG protein release by the cells of all donors increased with time from 9 to 48 h and was within the levels of the non-infected HPBCs. LPS stimulation, however, induced up-regulation of OPG when compared to the control cells in both models of infection (Supplementary Table 3).

**Table 4:** **Effect of *S. aureus* on OPG protein release by SM-HPBCs (white columns) and OM-HPBCs (gray columns)**.

**Acute infection**																								
**OPG**	**1 h**						**3 h**						**6 h**						**9 h**					
**pg.mL**−1	**Ctrl**		**10:1**		**30:1**		**Ctrl**		**10:1**		**30:1**		**Ctrl**		**10:1**		**30:1**		**Ctrl**		**10:1**		**30:1**	
Max	1705	2327	1639	3891	2053	3836	4933	8545	5813	6255	4800	8873	12,909	19,891	15,738	7227	12,773	17,636	16,133	22,065	15,147	18,370	15,413	23,043
Median	1308	1165	1128	1335	1181	1086	3365	3039	4646	2346	3268	3984	10,521	5634	10,817	6306	11,630	6654	4470	6162	5536	6401	5324	7288
Min	–	130	–	–	–	–	1000	826	1739	1522	–	2043	3565	3348	6391	4826	3565	5410	1443	3377	1902	5273	3475	4273
**Persistent infection**																								
**OPG**	**1 h**						**9 h**						**24 h**						**48 h**					
**pg.mL**−1	**Ctrl**		**10:1**		**30:1**		**Ctrl**		**10:1**		**30:1**		**Ctrl**		**10:1**		**30:1**		**Ctrl**		**10:1**		**30:1**	
Max	6719	6896	10,656	4938	9469	5688	17,469	12,646	10,625	10,902	11,844	9699	27,219	25,000	25,781	23,788	31,156	30,455	39,819	87,355	72,250	65,072	49,781	83,949
Median	1330	2346	1178	2529	1339	2539	4305	6915	6081	7108	5233	7253	11,548	13,958	12,679	16,361	13,515	18,292	21,231	33,149	26,050	27,990	24,734	30,538
Min	700	–	683	–	667	–	1834	155	2125	–	2334	–	4521	310	4979	143	4938	393	7875	1274	7021	1298	7083	1417

*Cells were exposed to viable S. aureus (MOI of 10:1 and 30:1) for 1, 3, 6, and 9 h in acute infection and for 1, 9, 24, and 48 h in persistent infection. Data are presented as minimal value, median value, and maximal value from four independent donors (– means undetermined)*.

To summarize, in the acute infection model, *S. aureus* induced a similar decrease in *OPG* mRNA expression in both SM- and OM-HPBCs, suggesting that HPBC maturation is not involved in such an effect. In our infection models, we also found that the OPG protein release by SM- and OM-HPBCs was not influenced by the presence of *S. aureus*, even if the cells of all our donors were responsive to LPS stimulation.

## Discussion

The bone microenvironment is a complex environment where osteoblasts and their precursors play a central role. Originating from the mesenchymal lineage, osteoblasts are the cells responsible for bone formation. Differentiation of bone-forming cells starts from mesenchymal stem cells, evolves through several states such as osteoprogenitors, pre-osteoblasts, and osteoblasts and ends up as matrix-embedded cells known as osteocytes (Aubin, 2001). Osteoblasts are the major cells for the synthesis of the bone organic matrix. The latter is mostly composed of type I collagen but also contains non-collagenous proteins such as fibronectin, osteopontin, and osteocalcin (Marie, 2008). Up to now, the conditioning culture media for osteoblast maturation *in vitro* was composed of ascorbic acid, β-glycerophosphate, and dexamethasone to trigger bone matrix mineralization (Park, 2012; Langenbach and Handschel, 2013). Here, for the first time to our knowledge, the maturation of bone cells with high alkaline phosphatase activity increased type I collagen fiber formation and the release of osseous proteins such as osteocalcin was obtained with a dexamethasone-free OM. Using both non-osteogenic and dexamethasone-free osteogenic media, we were able to establish two cell populations at different stages of maturation from the same patient, a step that is very important for assessing the effects of maturation on cell physiology without any genetic side effects. This is an important prerequisite as the impact of osteoblast maturation on *S. aureus* infection has not been taken into account to date. Furthermore, being able to obtain this maturation without the use of dexamethasone avoids its interference with the inflammatory response of HPBCs (Wright and Friedland, 2004), which is of great importance for the parameters studied in this paper.

Interaction between *S. aureus* and osteoblasts began to be documented with the demonstration of internalization by Hudson and colleagues. Then more recent studies highlighted the involvement of staphylococcal toxins and small colony variants during osteoblast infection (Hudson et al., 1995; Tuchscherr et al., 2015; Valour et al., 2015). Most of these studies that used live *S. aureus* focused on the persistent interaction with murine or human osteoblast cell lines, so the onset of infection and the consequences of extracellular growing bacteria were rarely considered except by Hamza and Li (2014).

Therefore, to document more precisely the effect of the degree of maturation of human osteoblasts on cell responses toward *S. aureus*, we used two different *in vitro* models of infection. The first one—the acute infection model—which allows direct interaction from 1 to 9 h between live *S. aureus* and HPBCs, was previously used to test the interaction of *S. aureus* with mesenchymal stem cells (Josse et al., 2014). This model examines the impact of both intracellular and extracellular staphylococci growing in the osteoblast environment. The second one—the persistent infection model—was adapted from a pre-existent *in vitro* model (Wright and Friedland, 2002), where extracellular staphylococci are specifically killed by gentamycin after 1 h of interaction in order to preserve only intracellular bacteria until 48 h.

Since infection of osteoblasts by *S. aureus* is a landmark in the development of osteomyelitis in bone tissue, we looked at various parameters that are associated with bacterial pathogenesis, such as attachment, internalization, bacterial survival, and the capacity to induce pro-inflammatory cytokines or chemokines that play a major role in the stimulation of other cells in the bone microenvironment. *S. aureus* NCTC 8325-4, a commonly used laboratory bacterium, was used to check whether the maturation of HPBCs can have an impact on its pathogenesis toward HPBCs and whether it can stimulate HPBCs as in previous early interaction cell models (Escotte et al., 2006; Al Alam et al., 2010).

Attachment is the first step in the bacterial infection process. SEM preparations of interactions of *S. aureus* on SM- and OM-HPBCs showed that *S. aureus* can bind to both types of cells with a greater susceptibility for OM-HPBCs, which could be due to a greater presence of extracellular matrix proteins around these HPBCs.

As seen previously by others, internalization is the prerequisite for the putative survival of *S. aureus* inside osteoblasts (Hudson et al., 1995; Tuchscherr and Löffler, 2016) and *S. aureus* collagen adhesion (Cna) and bone sialoprotein binding protein (Bbp) could play a synergistic role in the initial adhesion of *S. aureus* to osteoblasts, thereby promoting fibronectin-mediated internalization (Tung et al., 2000; Ahmed et al., 2001; Testoni et al., 2011). Comparing both models after 9 h of infection, it is nevertheless clear that extracellular bacteria were constantly internalized by the HPBCs, thus explaining why the intracellular pool is larger in the acute infection model than in the persistent one.

Furthermore, even if in our hands OM-HPBCs showed a higher expression of bone sialoprotein gene (*IBSP*) and a higher production of type I collagen fibers, no differences in internalization and survival of *S. aureus* inside SM- and OM-HPBCs could be seen, suggesting that *S. aureus* also uses mechanisms of internalization to invade HPBCs that are independent from the bone extracellular matrix.

Bone loss in osteomyelitis is due to the death of osteoblasts, a phenomenon that is itself potentiated by the increased activation of osteoclasts. A tight balance between bone formation by osteoblasts and bone resorption by osteoclasts is essential for the strength and integrity of bones. Therefore, we monitored the death of HPBCs after *S. aureus* infection, as well as the production of osteoclastic regulators.

Internalization of *S. aureus* is a key event in the induction of cell death through apoptosis (Bayles et al., 1998; Menzies and Kourteva, 1998; Kahl et al., 2000; Mempel et al., 2002). Here, as in a previous experiment with mesenchymal stem cells (Josse et al., 2014), cell death was observed after 6 h of acute infection, irrespective of HPBC maturation. Although Rasigade et al. recently reported that the activity of phenol-soluble modulins is the major factor in osteoblast killing by *S. aureus* and that α-hemolysin activity is negligible (Rasigade et al., 2013), the α-hemolysin produced by the extracellular *S. aureus* NCTC 8325-4 could play a role in the death phenomenon in acute infections, as no differences were noted in the persistent model where the bacteria were only intracellular.

Following interaction with *S. aureus*, the first line of defense for cells is to secrete inflammatory factors like cytokines or chemokines, which are able to activate and recruit immune cells in an attempt to clear the bacteria from the microenvironment (Turner et al., 2014).

For the first time, our study proposes a different kinetic of mRNA chemokine expression between infected SM- and OM-HPBCs as well as different profiles of expression in both the acute and persistent models.

In the acute infection model, *IL-6* mRNA over-expression by both SM- and OM-HPBCs infected with *S. aureus*, as well as the weak responses in *IL-1β* and *TNF-α* mRNA expressions, were already observed during *S. aureus* and mesenchymal stem cell interaction (Josse et al., 2014). In the persistent infection model, *IL-6* and *IL-1β* mRNA expression was increased after 48 h, as observed in *in vitro* models similar to ours (Bost et al., 1999; Marriott et al., 2002; Jauregui et al., 2013). Our results also suggest that cell maturation does not impact cytokine expression.

With regard to chemokines, while *CCL2* mRNA is significantly downregulated in the acute infection of OM-HPBCs after 9 h, it does not vary in SM-HPBCs. Moreover, concerning *CXCL1* and *CXCL8* mRNA expression, it is obvious that the SM-HPBCs are more susceptible to *S. aureus* than the OM-HPBCs in the acute model of infection. On the contrary, no difference in susceptibility was noted in the persistent infection model. *CCL2* and *CXCL1* mRNA expression was upregulated by OM-HPBCs after 24 and 48 h respectively whereas a significant over-expression of *CXCL8* mRNA by SM-HPBCs was observed after 48 h. Moreover, these responses were *S. aureus*-specific as LPS induced the same chemokine profiles on both SM-HPBCs and OM-HPBCs in our models. One explanation could be that immature SM-HPBCs are more susceptible to inducing acute inflammation and to recruiting neutrophils in response to extracellular *S. aureus* while OM-HPBCs, which are more involved in matrix formation and mineralization, might be involved in subsequent inflammation and could potentially recruit monocytes in response to intracellular bacteria. Furthermore, while *CCL2* and *CXCL8* mRNA expression increased by *S. aureus*-infected osteoblasts has already been observed in an *in vitro* model close to our model of persistent infection (Bost et al., 2001; Jauregui et al., 2013), this is the first time, to our knowledge, that an over-expression of *CXCL1* mRNA has been observed in *S. aureus*-infected HPBCs.

Bone-forming cells influence osteoclastogenesis through the production of RANKL and its decoy receptor OPG. OPG inhibits RANK/RANKL binding and thereby modulates the formation and activation of osteoclasts (Edwards and Mundy, 2011). In our persistent infection model, neither the expression of *RANKL* nor *OPG* mRNA varied, even in the presence of *S. aureus*. However, for the first time and in opposition to the increase in *OPG* expression after LPS stimulation, a decrease in *OPG* expression without any modulation of *RANKL* for either SM- or OM-HPBCs was noticed after 9 h in the acute infection model. This suggests that the regulation of *OPG* could be induced specifically by *S. aureus* and that extracellular bacteria could play a major role in the regulation.

Concerning the protein release of cytokines and chemokines, we did not observe any significant variations in the release by *S. aureus*-infected HPBCs compared to the non-infected counterparts whereas the upregulation of IL-6, CCL2, CXCL1, CXCL8, and OPG release occurred with LPS stimulation in both SM- and OM-HPBCs. These results are in opposition to early experiments where upregulation of IL-6, CCL2, and CXCL8 release was reported (Bost et al., 1999, 2001; Wright and Friedland, 2002; Jauregui et al., 2013) as well as a decrease in OPG release (Claro et al., 2011; Young et al., 2011). These differences could be due to conditions of the experimental procedures, notably the characteristics of the cells. In previous studies, *in vitro* experiments were mostly performed using osteoblastic cell lines or marketed normal osteoblasts coupled with high MOIs (75:1 or 250:1) to stimulate the cells. This may have led to an overestimation of the results. In our study, we used human primary cells from several independent donors. This certainly contributed to the wide inter-donor viability that we observed but it also strengthens our results with regard to the clinical outcome in patients.

In view of our findings, we suggest that osteoblasts are not the initiators of an inflammatory response but act more as reservoirs allowing the persistence of *S. aureus* inside bones and the development of chronic infections. Recent findings about small colony variants, staphylococcal toxin-related osteoblast killing, or the adaptation of *S. aureus* to chronicity provide further evidence for this hypothesis (Valour et al., 2015; Tuchscherr and Löffler, 2016; Tuchscherr et al., 2016).

## Conclusion

In this study, two models of *in vitro* interaction between *S. aureus* and HPBCs were established to investigate the impact of their maturation on osteomyelitis. HPBC maturation did not influence the internalization and survival of *S. aureus* inside HPBCs nor the cell death related to the infection. Furthermore, it had little impact on chemokine expression. Nevertheless, no variation in relative protein release was observed in the presence of *S. aureus*, suggesting the absence of any concrete inflammatory response by infected HPBCs. Our findings suggest that cell maturation does not impact the behavior of HPBCs infected with *S. aureus* and that the role of bone-forming cells (including osteoblasts) may not be pivotal for the inflammatory response in osteomyelitis.

## Author contributions

JJ, CG, CB, FL, CM, FD, and FV performed the experiments. JJ, FV, SG designed the research, analyzed the data, and wrote the paper.

## Funding

JJ is the recipient of a fellowship from the French ≪ Ministère de l'Enseignement Supérieur et de la Recherche ≫. This work was partially supported by a grant from the University of Reims Champagne Ardenne (BQR, 2011).

### Conflict of interest statement

The authors declare that the research was conducted in the absence of any commercial or financial relationships that could be construed as a potential conflict of interest.
