# Supplementary material for: Impact of the Maturation of Human Primary Bone-Forming Cells on Their Behavior in Acute or Persistent Staphylococcus aureus Infection Models
Source: Front Cell Infect Microbiol. 2016 Jun 21;6:64. doi: 10.3389/fcimb.2016.00064 (PMC4914565; doi:10.3389/fcimb.2016.00064)
Supplement: Supplementary file 1 [file DataSheet2.docx]

Supplementary Material

**Impact of the maturation of human primary bone-forming cells on their behavior in acute or persistent *Staphylococcus aureus* infection models**

**Jérôme Josse^1,2^, Christine Guillaume^1,3^, Camille Bour^1^, Flora Lemaire^1^, Céline Mongaret^1,2^, Florence Draux^1,3^, Frédéric Velard^1,3^ and Sophie C. Gangloff^1,2*^**

^1^EA 4691 « Biomatériaux et inflammation en site osseux », Pôle Santé, Université de Reims Champagne-Ardenne, Reims, France

^2^UFR Pharmacie, Pôle Santé, Université de Reims Champagne-Ardenne, Reims, France

^3^UFR Odontologie, Pôle Santé, Université de Reims Champagne-Ardenne, Reims, France

*** Correspondence:**Prof. Sophie GANGLOFF,

EA 4691 Biomatériaux et inflammation en site osseux, UFR de Pharmacie de Reims,

1 avenue du Maréchal Juin, 51095 Reims Cedex, France

Email: [sophie.gangloff@univ-reims.fr](mailto:sophie.gangloff@univ-reims.fr)

**
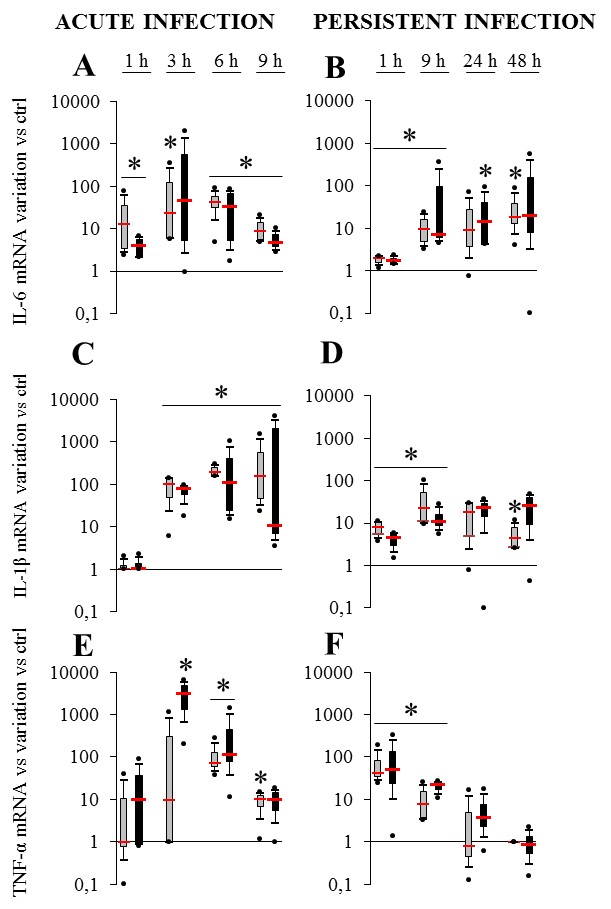
**

**Supplementary Figure 1:** **Effect of LPS stimulation on *IL-6* (A, B), *IL-1β* (C, D) and *TNF-α* (E, F) mRNAs expressions by SM-HPBCs (grey boxes) and OM-HPBCs (black boxes).** Cells were stimulated with LPS (10 µg.mL^-1^) for 1 h, 3 h, 6 h and 9 h in acute infection (**A**, **C, E**) and for 1 h, 9 h, 24 h and 48 h in persistent infection (**B**, **D, F**). Expressions of *IL-6,* *IL-1β* and *TNF-α* mRNA were evaluated by RT-PCR analysis. Data are shown as specific variation of mRNA compared to relative non-infected / non-stimulated cells using the 2^-ΔΔCt^ method (*HPRT-1* was used as internal control). * means p < 0.05 vs relative non-infected / non-stimulated cells for each time period. Experiments were performed on 4 independent donors.


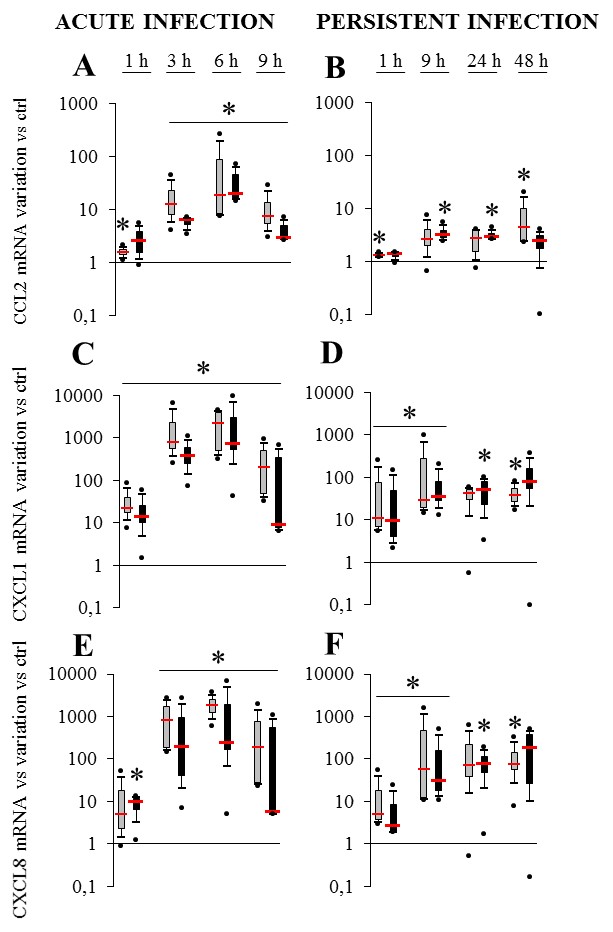


**Supplementary Figure 2:** **Effect of LPS stimulation on *CCL2* (A, B), *CXCL1* (C, D) and *CXCL8* (E, F) mRNAs expressions by SM-HPBCs (grey boxes) and OM-HPBCs (black boxes).** Cells were stimulated with LPS (10 µg.mL^-1^) for 1 h, 3 h, 6 h and 9 h in acute infection (**A**, **C, E**) and for 1 h, 9 h, 24 h and 48 h in persistent infection (**B**, **D, F**). Expressions of *CCL2*, *CXCL1* and *CXCL8* mRNA were evaluated by RT-PCR analysis. Data are shown as specific variation of mRNA compared to relative non-infected / non-stimulated cells using the 2^-ΔΔCt^ method (*HPRT-1* was used as internal control). * means p < 0.05 vs relative non-infected / non-stimulated cells for each time period. Experiments were performed on 4 independent donors.


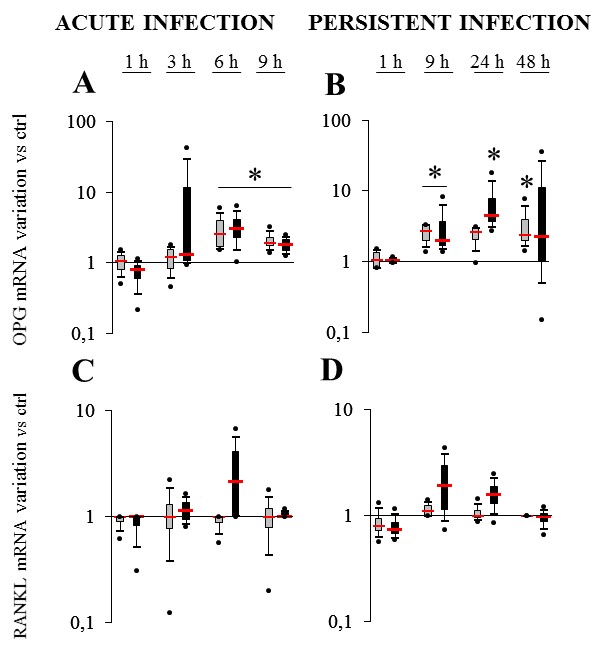


**Supplementary Figure 3:** **Effect of LPS stimulation on *OPG* (A, B) and *RANKL* (C, D) mRNAs expressions by SM-HPBCs (grey boxes) and OM-HPBCs (black boxes).** Cells were stimulated with LPS (10 µg.mL^-1^) for 1 h, 3 h, 6 h and 9 h in acute infection (**A**, **C, E**) and for 1 h , 9 h, 24 h and 48 h in persistent infection (**B**, **D, F**). Expressions of *OPG* and *RANKL* mRNA were evaluated by RT-PCR analysis. Data are shown as specific variation of mRNA compared to relative non-infected / non-stimulated cells using the 2^-ΔΔCt^ method (*HPRT-1* was used as internal control). * means p < 0.05 vs relative non-infected / non-stimulated cells for each time period. Experiments were performed on 4 independent donors.

**Supplementary Table 1:** **Effect of LPS stimulation on IL-6 protein release by SM-HPBCs (white columns) and OM-HPBCs (grey columns).** Cells were stimulated with LPS for 1 h, 3 h, 6 h and 9 h in acute infection and for 1 h and additional incubations to reach 9 h, 24 h and 48 h in persistent infection. Data are presented as minimal value, median value and maximal value from 4 independent donors (- means undetermined).

**Supplementary Table 2:** **Effect of LPS stimulation on CCL2, CXCL1 and CXCL8 protein releases by SM-HPBCs (white columns) and OM-HPBCs (grey columns).** Cells were stimulated with LPS for 1 h, 3 h, 6 h and 9 h in acute infection and for 1 h and additional incubations to reach 9 h, 24 h and 48 h in persistent infection. Data are presented as minimal value, median value and maximal value from 4 independent donors (- means undetermined).

**Supplementary Table 3:** **Effect of LPS stimulation on OPG protein release by SM-HPBCs (white columns) and OM-HPBCs (grey columns).** Cells were stimulated with LPS for 1 h, 3 h, 6 h and 9 h in acute infection and for 1 h and additional incubations to reach 9 h, 24 h and 48 h in persistent infection. Data are presented as minimal value, median value and maximal value from 4 independent donors (- means undetermined).
